# Oculomotor Deficits and Symptom Severity Are Associated With Poorer Dynamic Mobility in Chronic Mild Traumatic Brain Injury

**DOI:** 10.3389/fneur.2021.642457

**Published:** 2021-07-26

**Authors:** Linda J. D'Silva, Prabhakar Chalise, Sakher Obaidat, Michael Rippee, Hannes Devos

**Affiliations:** ^1^Department of Physical Therapy, Rehabilitation Science, and Athletic Training, University of Kansas Medical Center, Kansas City, MO, United States; ^2^Department of Biostatistics and Data Science, University of Kansas Medical Center, Kansas City, MO, United States; ^3^Department of Neurology, University of Kansas Health System, Kansas City, MO, United States

**Keywords:** baseline visual acuity, post-concussion symptom scale, dizziness handicap inventory, functional gait assessment, chronic mild traumatic brain injury

## Abstract

Oculomotor deficits, vestibular impairments, and persistent symptoms are common after a mild traumatic brain injury (mTBI); however, the relationship between visual-vestibular deficits, symptom severity, and dynamic mobility tasks is unclear. Twenty-three individuals (mean age 55.7 ± 9.3 years) with persistent symptoms after mTBI, who were between 3 months to 2 years post-injury were compared with 23 age and sex-matched controls. Oculomotor deficits [depth perception, near-point convergence, baseline visual acuity (BLVA), perception time], vestibular deficits (dynamic visual acuity in the pitch and yaw planes), dynamic mobility measured by the Functional Gait Assessment (FGA), and symptoms measured by the Post-Concussion Symptom Scale (PCSS) and Dizziness Handicap Inventory (DHI) were compared between groups. Participants with mTBI had poorer performance on the FGA (*p* < 0.001), higher symptom severity on the PCSS (*p* < 0.001), and higher DHI scores (*p* < 0.001) compared to controls. Significant differences were seen on specific items of the FGA between individuals with mTBI and controls during walking with horizontal head turns (*p* = 0.002), walking with vertical head tilts (*p* < 0.001), walking with eyes closed (*p* = 0.003), and stair climbing (*p* = 0.001). FGA performance was correlated with weeks since concussion (*r* = −0.67, *p* < 0.001), depth perception (*r* = −0.5348, *p* < 0.001), near point convergence (*r* = −0.4717, *p* = 0.001), baseline visual acuity (*r* = −0.4435, *p* = 0.002); as well as with symptoms on the PCSS (*r* = −0.668, *p* < 0.001), and DHI (*r* = −0.811, *p* < 0.001). Dynamic balance deficits persist in chronic mTBI and may be addressed using multifaceted rehabilitation strategies to address oculomotor dysfunction, post-concussion symptoms, and perception of handicap due to dizziness.

## Introduction

Mild traumatic brain injury (mTBI) is challenging to diagnose and manage because of the widely disparate mechanisms of injury, a range of associated symptoms, and a spectrum of recovery trajectories (1–3). In the United States alone, 75% of the estimated 2.5–3.8 million people who sustain a traumatic brain injury (TBI) annually, are considered mild (4, 5). Diagnosis of mTBI in middle-aged and older adults is further complicated due to pre-existing comorbidities, side-effects of medications, and many physical and psychosocial factors (6–8). As a result many concussive events remain unrecognized and untreated (9), which may have work-related consequences for middle-aged adults (10), and could lead to decline in function (11, 12) and potential loss of independence for older adults (13).

In some individuals with mTBI, symptoms persist, evolve, or new symptoms develop beyond the 3-month period since injury; (1, 14–16) these individuals in the chronic stage of mTBI are said to have “post-concussion syndrome” (16). The number of people with post-concussion syndrome is unclear, with prior studies reporting numbers ranging from 15 to 82% (3, 9, 15, 17). Oculomotor (18), vestibular (19, 20), and visual-vestibular processing deficits (21–23) have been reported in the chronic stage of mTBI. These deficits may arise from injury to the peripheral vestibular structures (11, 24, 25) or due to traumatic central axonal injury (26–30). Several studies have examined adults in the chronic stage of mTBI showing higher post-concussion symptom severity (15, 31–33), poorer balance control (23, 34), reduced frequency and intensity of leisure activities (13), change in activity and driving habits (10, 35), and lower quality of life (15). Skóra et al. (20) report persistent central vestibular dysfunction in their cohort of middle-aged participants (mean age 44.4 ± 13.6 years) 6 months after mTBI, although symptoms of dizziness had decreased in intensity. Using inertial sensors to capture gait and turning dynamics, Fino et al. (36) have reported that their participants (mean age 38.4 ± 9.9 years) with chronic mTBI with peripheral vestibular or oculomotor dysfunction had slower turning speed compared to healthy controls which were associated with higher severity of post-concussive symptoms, while Martini et al. (37) report that single task and dual-task gait are altered in persons with chronic mTBI (mean age 39.6 ± 11.7 years). Row et al. (23) report that in their cohort (mean age 47.49 ± 16.12 years) vestibular and motor control deficits are associated with higher symptoms of dizziness. These studies have examined specific aspects of gait, such as turning dynamics, gait variability, rhythm, and motor control measured by accelerometers or laboratory equipment that may not be easily accessible to clinician practitioners. Hence, the purpose of this study was to examine the relationship between visual-vestibular deficits, symptom severity, and a clinical test that examines dynamic balance.

The Functional Gait Assessment (FGA) is a clinical test that assesses dynamic balance control during daily activities such as walking with head turns, stepping over objects, walking in the dark among others (38, 39). The purpose of this pilot study was to examine the relationship between visual dysfunction, peripheral vestibular deficits, symptoms quantified by the post-concussion symptom scale and dizziness handicap inventory, and functional mobility in adults between 40 and 80 years of age who were between 3 months and 2 years post-injury. Our hypotheses were (1) Visual deficits (measured by depth perception, near-point convergence, baseline visual acuity, perception time), and peripheral vestibular deficits (measured by the dynamic visual acuity test) will be associated with poorer performance on the FGA; (2) Higher symptom severity on the Post-Concussion Symptom Scale (PCSS) and higher perception of handicap due to dizziness on the Dizziness Handicap Inventory (DHI) will be associated with poorer performance on the FGA.

## Materials and Methods

### Study Design

This was a cross-sectional, comparative study conducted at the University of Kansas Medical Center. The study protocol was approved by the University's Institutional Review Board.

### Participants

Participants with mTBI were recruited from the Neurology clinic, with the assistance of a neurologist (MR). Additionally, the Healthcare Enterprise Repository for Ontological Narration (HERON) (40, 41) search discovery tool was used to identify persons with mTBI who were seen at the University hospital and who met inclusion and exclusion criteria. Participants who met screening criteria were contacted if they had signed up for the Pioneers Research participant registry. Participants were included if they were: (1) Between 40 and 80 years of age, (2) Had a diagnosis of mTBI coded by ICD-10 codes (S06.0X0A–S06.0X9S) or F07.81, (3) Had persistent symptoms from their injury (determined with a subjective self-report), (4) Were between 3 months and 2 years since their injury. Due to the exploratory nature of the study, we included a broad age range. We selected 3 months to 2 years as the time frame since injury. The 3-month period allows for spontaneous recovery after mTBI, while the 2-year period was chosen based on the patient population that comes to the neurology clinic.

Participants were excluded if they (1) Had a diagnosed neurological problem, or a history of a visual or vestibular disorder prior to the mTBI, (2) Had lower extremity injury, recent surgery or pain that would impact the walking tests, (3) Had a history of cancer and received chemotherapy, or (4) If they were involved in litigation due to the injury. Exclusion criteria were based on evidence that chemotherapy can independently affect the peripheral vestibular system while persons involved in litigation have higher stress levels that could affect performance (42–44).

Healthy controls with no history of head injury were recruited through word-of-mouth from the campus, and from the community, and were individually matched for sex and age (±5 years).

### Study Procedure

Participant eligibility was verified using a phone screen and eligible participants were scheduled for a testing session. All participants were informed to wear comfortable shoes and bring their corrective eyewear to the testing session. After completing informed consent, demographic information, medical history such as height, weight, medication list; and for people with mTBI, the date of injury was collected. Systems review included history of hearing disorders, migraines, and comorbidities. A clinical examination was completed (LD) which included sensory testing, motor testing, and positional testing to rule out benign paroxysmal positional vertigo (BPPV). Near point convergence was tested using the Royal Air force convergence ruler and depth perception was tested using the Randot stereo test (45). Following the systems review and clinical assessments, the tests and questionnaires below were completed in random order based on a random number generator.

The Bertec® Vision Advantage™ (Bertec® Corporation, Columbus, Ohio, USA) was used to administer the Dynamic Visual Acuity Test (DVAT). It includes a wireless inertial measurement unit mounted in the center of the participant's forehead using an elastic headband with a 3-axis integrating gyro (Yost 3-Space Wireless Sensor, Yost Labs) to determine rotational head velocity in the yaw and pitch planes (46). Similar to the work of Quintana et al., the inertial measurement unit was used to quantify velocity and identify direction of head movement, with a sampling frequency of 175 Hz. The measures of visual acuity were recorded in Logarithm of the Minimum Angle of Resolution (LogMAR) units. The LogMAR value of zero corresponds to 20/20 which is the standard for normal vision. The lower the LogMAR, the better the visual acuity. A negative LogMAR unit represents better than 20/20 vision.

Participants were seated in a study chair, five feet from the computer screen. The testing procedure required two baseline assessments which included testing of baseline visual acuity (BLVA) and perception time test (PTT) to individualize the dynamic assessments to the person being tested. BLVA was measured with the person's head stationary where an optotype (the letter “E”) was presented on the computer screen and they had to correctly identify the orientation of the “E.” Based on the Hughson-Westlake algorithm, the smallest optotype that was correctly identified 66% of the time, was considered their BLVA. For PTT, the optotype appeared for varying time periods in milliseconds to determine the time that the participant required to perceive the optotype. For the DVAT, participants had to generate active rotational head movements to 20 degrees from midline in each direction at a target velocity of 100 degrees per second (with a range from 85 to 120 degrees/second). Once the participant's achieved the desired head velocity, the optotype “E” appeared in the center of the screen. DVAT was recorded as the size of the smallest optotype that the participant could identify while rotating the head at or above the minimum velocity. For the pitch plane, the participant had to achieve a target head velocity of 80 degrees/second (range from 60 to 100 degrees/second) for the optotype to appear. The participants were set up to complete 15 trials in each direction. The outcome variables for the DVAT was loss of lines in logMAR value to the right and left in the yaw plane, and up and down in the pitch plane. Higher logMAR values indicate greater loss of dynamic visual acuity (25, 47–49).

Participants completed the FGA which consists of mobility tasks in 10 conditions such as walking with head turns and tilts, walking with eyes closed, pivot turns, and stepping over obstacles among others. Participants walked on a 20-foot walkway that was 12 inches wide and were scored on a Likert scale from 0 to 3, where 0 indicated severe impairment and 3 was normal. A score of 30 indicates a perfect score with no mobility impairments. The FGA has been validated in persons with vestibular disorders and scores of 22 or less indicate high risk for falls (38, 39). The FGA was administered by a single physical therapist (LD), it has a high intra-rater reliability with an intraclass correlation coefficient of 0.99 (95% CI: 0.97–0.99) (50).

The subjective questionnaires were completed after instructions were provided by the investigators. Participants completed the PCSS which contains 22 self-reported symptoms that can be rated on a 7-point Likert scale with zero indicating “none” and six indicating “severe” complaint. The maximum PCSS score is 132 with higher scores reflecting either more symptoms or higher severity of fewer symptoms (51, 52). The PCSS is divided into somatic, cognitive, emotional, and sleep sub-scales (53). The DHI is a measure of self-reported activity and participation restrictions due to either dizziness or unsteadiness with a maximum score of 100 points (54, 55). Participants were asked to score how dizziness or imbalance may affect their participation on each item. The DHI is further divided into physical, emotional, and functional sub-domains. People with scores of more than 60 points on the DHI have been shown to have a higher risk of falls (56).

### Statistical Analysis

Data were inspected for normality using histograms and the Kolmogorov–Smirnov test of normality. Independent sample *t*-tests were used to compare data that was normally distributed (age, BMI, DVA loss upward, downward, right, and left in LogMAR), while data that was not normally distributed were compared using Mann–Whitney *U* (depth perception, near point convergence, baseline visual acuity, processing time, FGA score, PCSS score and sub scales, and DHI score and sub scales). Levene's test was examined to determine equality of variance. Scores on each item of the FGA were compared between groups using Mann–Whitney *U*-tests. Alpha was set a priori to 0.05, but Bonferroni's correction was applied for the independent variables (visual, vestibular, symptoms; total of 10), and the individual items on the FGA test (total of 10); therefore, alpha was set to 0.005 for these group analyses. Spearman's rank correlation coefficients were used to assess the relationship between FGA score and each independent variable (14 variables in total, with Bonferroni adjustment alpha was set at 0.004). Furthermore, correlations were examined between FGA score and each subscale of the DHI and PCSS. Correlations were interpreted as fair (0.25–0.50), moderate (0.5–0.75), and good (>0.75). All analyses were conducted using SPSS for Windows version 25.0 (SPSS Inc., Chicago, USA).

## Results

**Figure 1:**
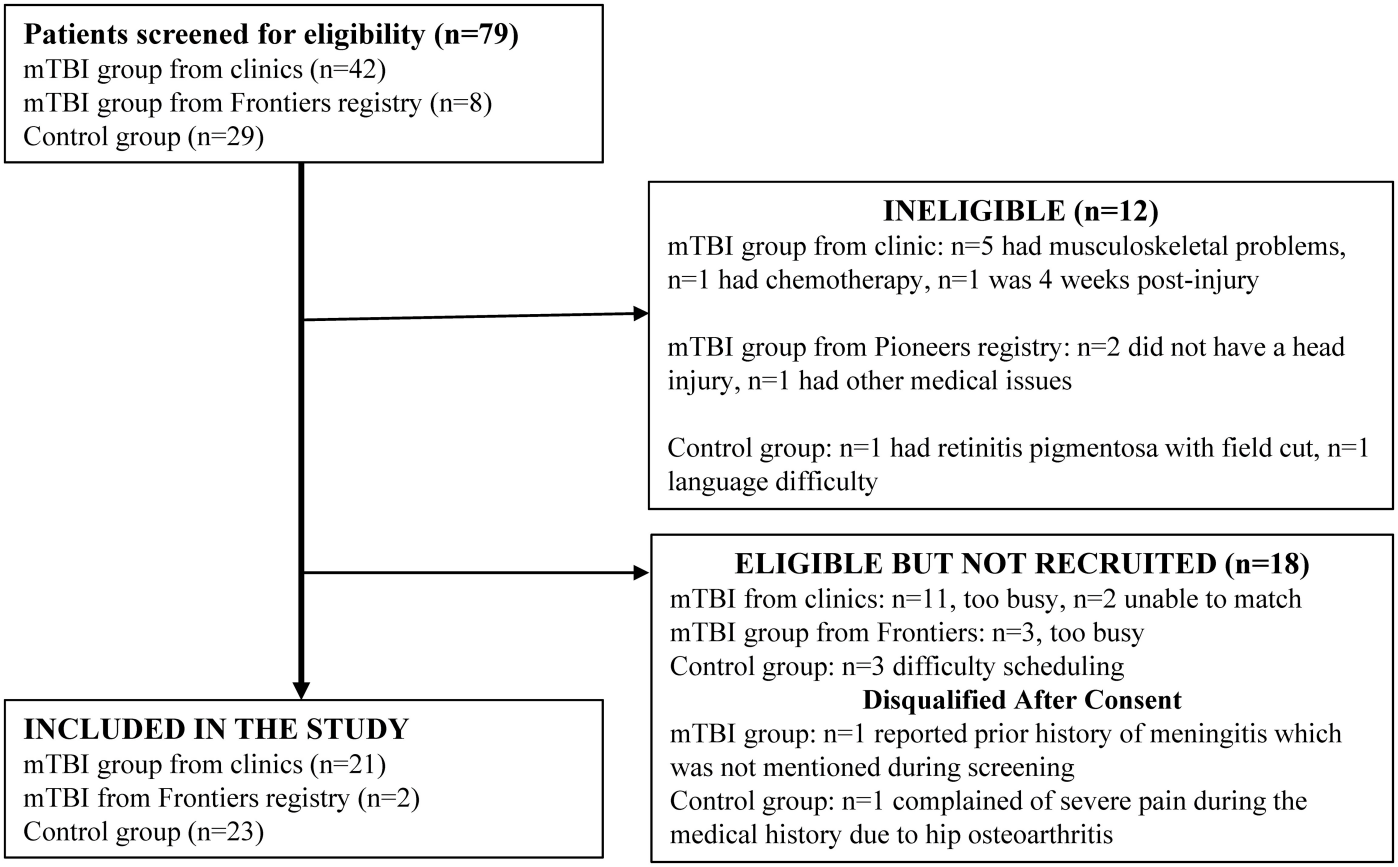
Participant recruitment with reasons for exclusion.

Forty-two individuals with mTBI were screened through the neurology clinic at the University of Kansas Hospital. Thirty-two individuals were identified through the HERON database search and Pioneers Registry, however only eight were contactable. Twenty-nine controls were screened from the campus and community. The participant flow diagram depicts subjects screened, enrolled, and excluded (Figure 1).

### Participant Characteristics

Forty-six individuals completed the study; 23 in the mTBI group (19 females and 4 males) and 23 age and sex-matched controls. The mean duration since injury was 33.2 ± 5.1 weeks (range: 12–92 weeks). Three control subjects had diagnoses of hearing loss (two were genetic) and three had a prior history of migraines. In the mTBI group, two participants complained of tinnitus since the injury, two had a prior history of migraines, and three were wearing prescription glasses with prisms. No strength deficits were noted with manual muscle testing, sensation in the feet was impaired in one control and two persons with mTBI, none of the participants had BPPV.

### Comparisons Between mTBI and Control Groups

**Figure 2:**
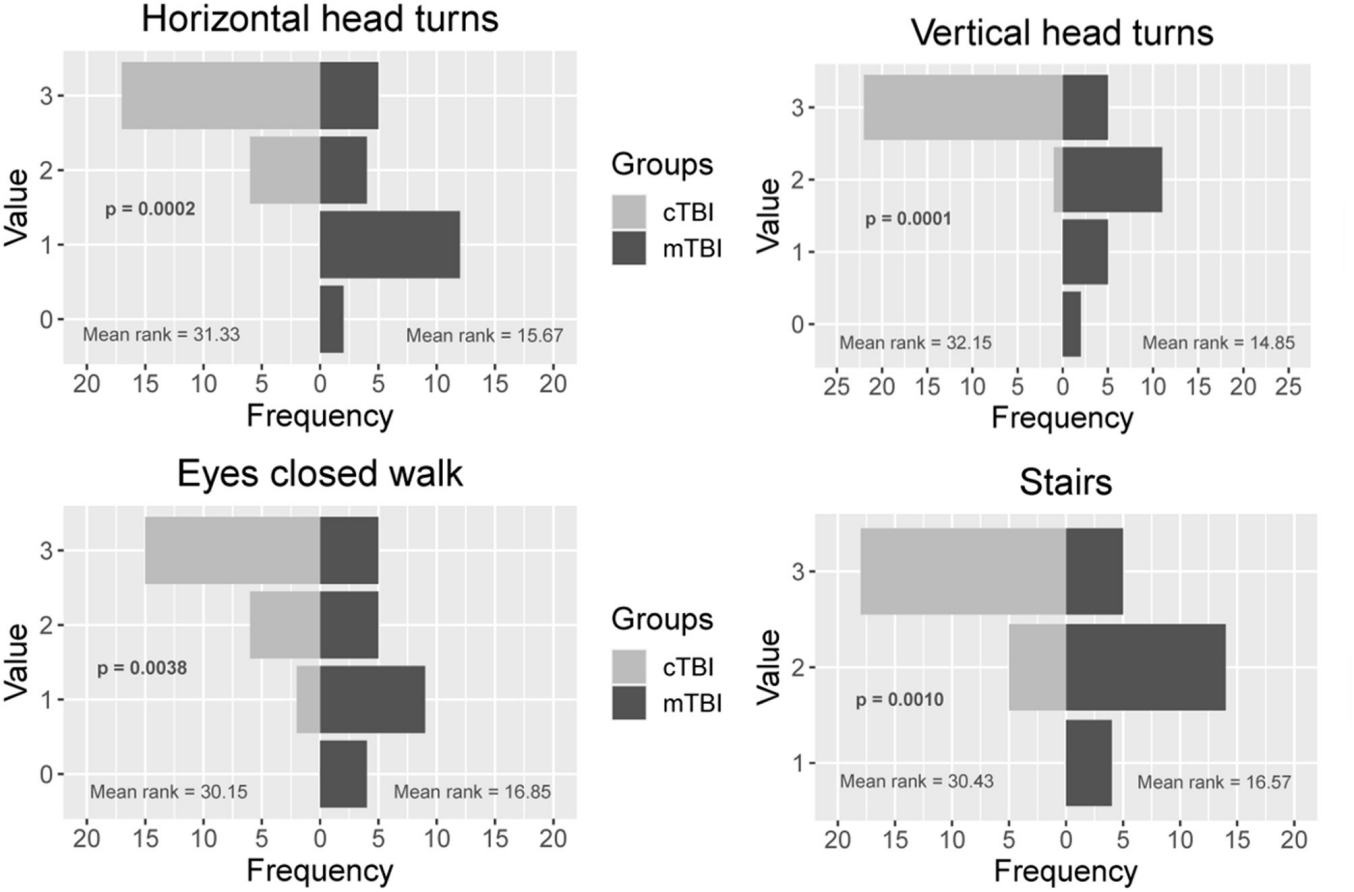
Comparison between the control and mTBI group on 4 individual items of the Functional Gait Assessment. Values range from 0 to 3 where 3 = normal performance, 1 = mild impairment, 2 = moderate impairment, and 0 = severe impairment. mTBI, mild traumatic brain injury.

Participants with mTBI had poorer performance on the FGA (*p* < 0.001), higher severity of symptoms on the PCSS (*p* < 0.001), and higher perception of handicap due to dizziness (*p* < 0.001) compared to controls (Table 1). Significant differences between participants with mTBI and controls were seen in near point convergence (*p* = 0.003), while none of the dynamic visual acuity measures were significantly different between groups (Table 2). Examination of the 10 individual items of the FGA, showed significant differences between participants with mTBI and controls during walking with horizontal head turns (*p* = 0.002), walking with vertical head tilts (*p* < 0.001), walking with eyes closed (*p* = 0.003), and stair climbing (*p* = 0.001; Figure 2).

**Table 1:** Participant demographics.

	**mTBI group**	**Control group**	***p*-value**
	**(*n* = 23)**	**(*n* = 23)**	
Age (years)^a^ (mean ± SD)	55.70 ± 9.3	55.13 ± 9.1	*p* = 0.84
Sex (Female/male)	19/4	19/4	
BMI (kg/m^2^)^a^ (mean ± SD)	31.4 ± 7.9	28.77 ± 6.5	*p* = 0.22
Weeks since injury	33.23 ± 5.1	NA	
Post-concussion Symptom Scale^b^ (median, range)	58.50 (9–110)	2 (0–37)	*p* < 0.001^*^
Dizziness Handicap Inventory^b^ (median, range)	54 (10–90)	0 (0–2)	*p* < 0.001^*^
Functional Gait Assessment^b^ (median, range)	22 (7–29)	29 (22–30)	*p* < 0.001^*^

a *Indicates comparisons using independent t-tests*,

b *indicates comparison of distributions using Mann–Whitney U-test*.

* *indicates significant differences between groups (Bonferroni < 0.005). BMI, body mass index; mTBI, mild traumatic brain injury*.

**Table 2:** Differences between the mTBI and control groups in visual and vestibular outcomes.

	**mTBI group**	**Control group**	***p*-value**
Depth perception^a^	70 (60)	30 (50)	*p* = 0.04
(median, IQR, 95% CI)	(50.03, 125.63)	(28.56, 105.35)	
Near point convergence (cm)^a^	22 (24)	10 (17)	*p* = 0.003^*^
(median, IQR, 95% CI)	(18.98, 31.37)	(9.92, 17.65)	
Baseline visual acuity (LogMAR)^a^	−0.07 (0.22)	−0.11 (0.10)	*p* = 0.15
(median, IQR, 95% CI)	(−0.09, 0.02)	(−0.16, −0.05)	
Perception time (ms)^a^	30 (2.5)	30 (0)	*p* = 0.32
(median, IQR, 95% CI)	(30.38, 35.27)	(29.25, 34.64)	
Downward DVA loss (LogMAR)^b^	0.22 ± 0.11	0.22 ± 0.10	*p* = 1.0
(Mean, SD, 95% CI)	(0.17, 0.26)	(0.17, 0.26)	
Upward DVA loss (LogMAR)^b^	0.24 ± 0.12	0.19 ± 0.09	*p* = 0.18
(Mean, SD, 95% CI)	(0.18, 0.29)	(0.16, 0.23)	
Right DVA loss (LogMAR)^b^	0.21 ± 0.11	0.20 ± 0.09	*p* = 0.78
(Mean, SD, 95% CI)	(0.17, 0.26)	(0.16, 0.24)	
Left DVA loss (LogMAR)^b^	0.21 ± 0.09	0.21 ± 0.11	*p* = 0.98
(Mean, SD, 95% CI)	(0.18, 0.25)	(0.17, 0.26)	

a *indicates comparisons using the Mann–Whitney U-test*.

b *indicates comparisons using the independent samples t-test*.

* *indicates significant differences between groups (Bonferroni < 0.005). mTBI, mild traumatic brain injury; DVA, dynamic visual acuity*.

### Correlation Analyses Between Oculomotor Deficits, Symptoms, and FGA Performance

**Figure 3:**
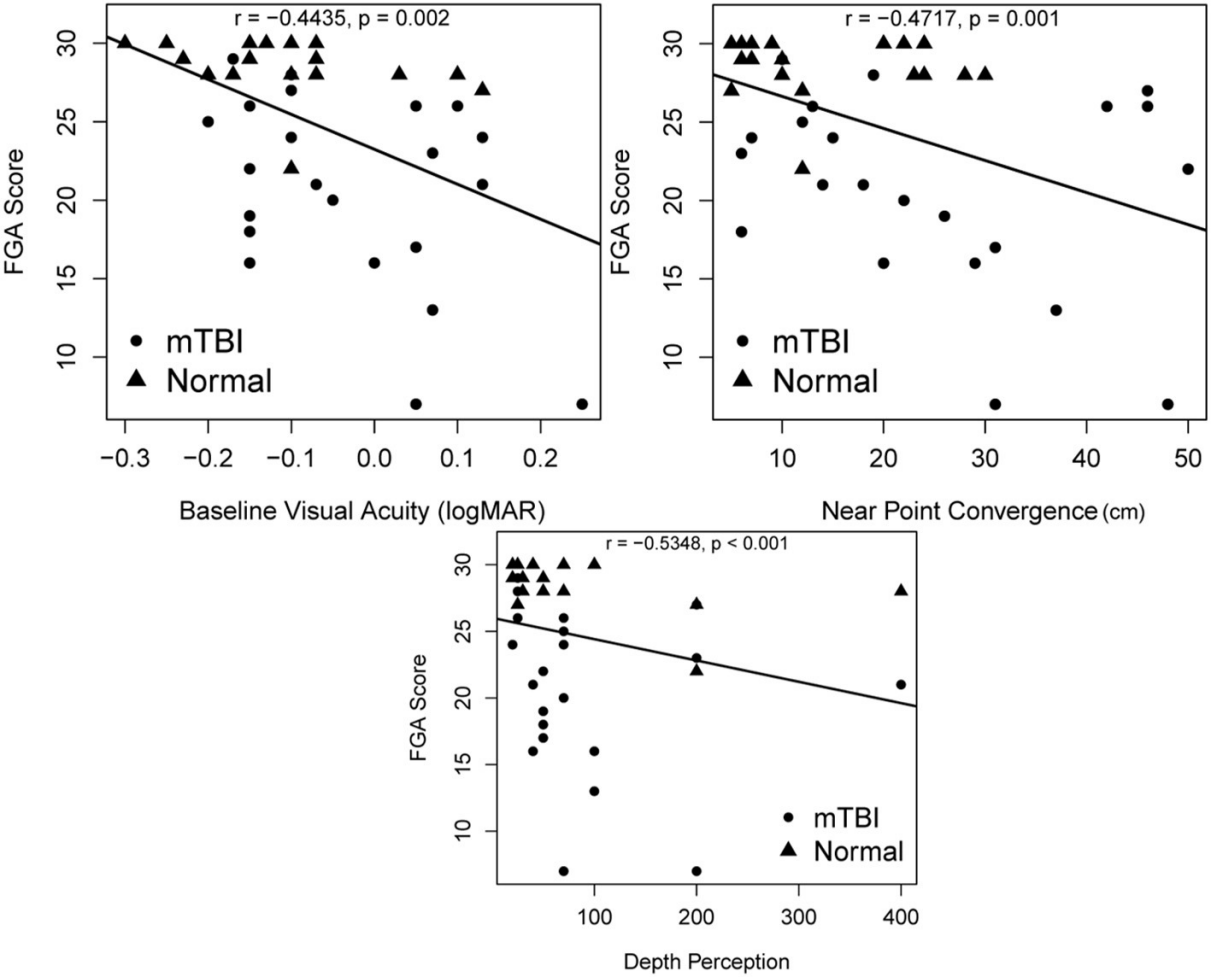
Associations between FGA scores and oculomotor deficits including depth perception, near point convergence, and baseline visual acuity. *p*-values are significant at α = 0.004 after Bonferroni adjustment. FGA, Functional Gait Assessment.

**Figure 4:**
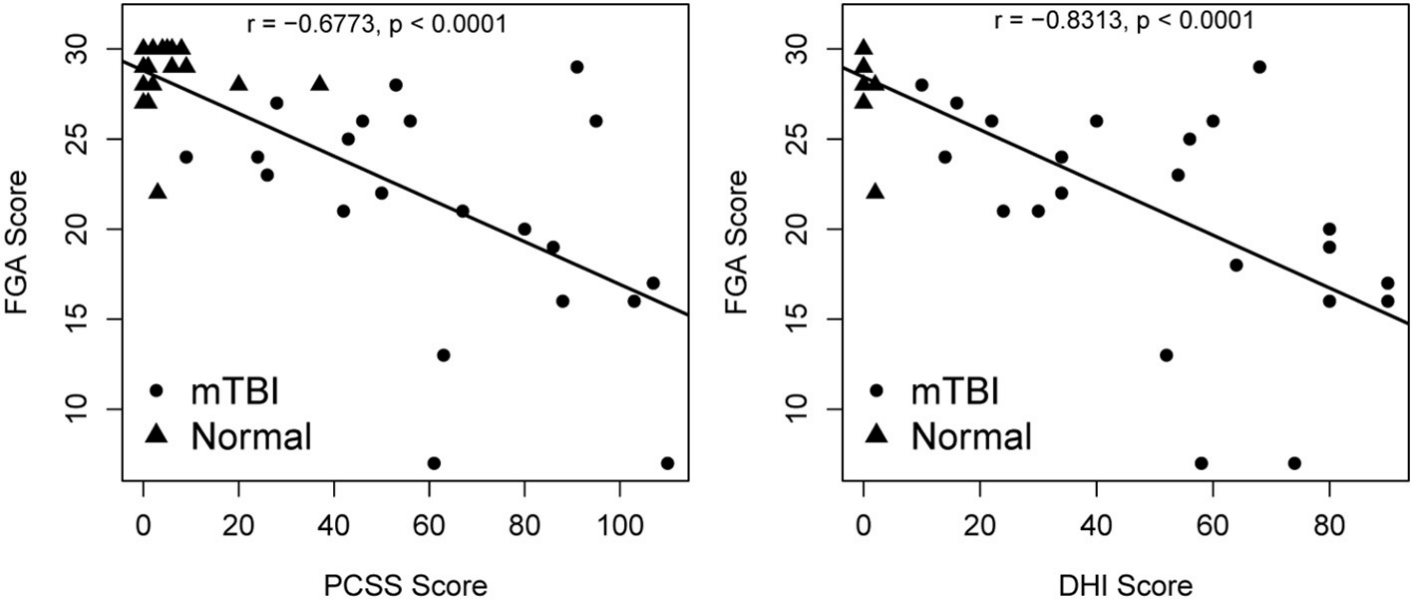
Relationship between DHI, PCSS, and FGA score. *p*-values are significant at α = 0.004 after Bonferroni adjustment. DHI, Dizziness Handicap Inventory; PCSS, Post-concussion Symptom Scale; FGA, Functional Gait Assessment.

Fair to moderate correlations were noted between FGA score and weeks since concussion (*r* = −0.67, *p* < 0.001), depth perception (*r* = −0.5348, *p* < 0.001), near point convergence (*r* = −0.4717, *p* = 0.001), baseline visual acuity (*r* = −0.4435, *p* = 0.002) (Figure 3), and PCSS score (*r* = −0.668, *p* < 0.001) while good correlation were noted with the DHI score (*r* = −0.811, *p* < 0.001) (Figure 4).

The DHI was examined by the three subscales between the mTBI and control groups, physical (16.1 vs. 0, *p* < 0.001), emotional (15.2 vs. 0, *p* < 0.001), and functional (19.13 vs. 0.17, *p* < 0.001). Significant associations between FGA score and physical subscale (*r* = −0.77, *p* < 0.001), emotional subscale (*r* = 0.79, *p* < 0.001), and functional subscale (*r* = −0.81, *p* < 0.001) were observed. Highest symptom provocation on the physical subscale were reported by 56% of people with mTBI during “ambitious activities such as sports, dancing, and household chores,” and by 52% while “doing quick head movements.” Within the functional subscale, 48% of participants with mTBI reported that they had difficulty performing strenuous activity, while 43% had difficulty walking around the house in the dark. Within the emotional subscale, 43% reported feeling frustrated, and having difficulty concentrating.

The PCSS was examined based on subscales between the mTBI and control groups, somatic (27.3 vs. 1.3, *p* < 0.001), cognitive (13.86 vs. 1.3, *p* < 0.001), sleep (9.8 vs. 1.3, *p* < 0.001) and emotional (10.6 vs. 1.0, *p* < 0.002). Significant associations were observed between FGA score and somatic symptoms (*r* = −0.72, *p* < 0.001), cognitive symptoms (*r* = −0.72, *p* < 0.001), sleep subscale (*r* = −0.60, *p* < 0.001), and emotional symptoms (*r* = −0.61, *p* < 0.001).

## Discussion

Results of this study show that adults (mean age of 55.7 ± 9.3 years) who had persistent symptoms after a mTBI had poorer dynamic mobility scores on the FGA. Oculomotor deficits, higher post-concussion symptoms, and higher perception of handicap due to symptoms of dizziness or imbalance were associated with poorer dynamic balance on the FGA test.

More than 30% of individuals with mTBI experience feeling off-balance, even though this feeling of imbalance is not easily detectable (11, 57). Functional mobility relies on the central nervous system to combine and transform sensory information seamlessly into a motor output (58, 59). Slower gait speed (9), poorer static balance control (23), altered gait dynamics during turning activities (36), and impaired motor control at the limits of stability (23), have been described in chronic mTBI. The current study has extended previous study findings to examine dynamic balance during the performance of daily mobility activities. Forty-eight percent of the participants with mTBI in our study had FGA scores below 22, indicating poor dynamic functional mobility. Oculomotor abnormalities such as poorer depth perception, poorer baseline visual acuity, longer near point convergence distance were moderately correlated with poorer functional mobility. Visual deficits have been studied extensively after traumatic brain injury (45, 60–66), and in combination with vestibular dysfunction have been shown to affect balance (21). Kleffelgaard et al. examined 65 individuals (mean age 39.2 ± 12.9 years) who were 3 months post-injury and reported that 78% had poorer high-level balance while 77% had lower scores on high level mobility tasks compared to normative values. Of these individuals 62% had positive findings during oculomotor tests while 29% had positive findings with the dynamic visual acuity test (57). Results of our study show that the tasks on the FGA that were most difficult for people with mTBI compared to healthy controls were walking with horizontal head turns, vertical head tilts, tandem walking, stepping over an obstacle, walking with eyes closed, and stair climbing. These findings are similar to those reported by Chou et al. (12) who have shown higher medio-lateral instability in people with mTBI (mean age 40.9 ± 11.3 years), which is increased while navigating obstacles in the environment. Fino et al. performed clinical tests of oculomotor function and examined the peripheral vestibular system in 14 adults with chronic mTBI (mean age 38.4 ± 9.9). They report that all participants had evidence of oculomotor or peripheral vestibular dysfunction on at least one clinical test which was associated with slower turning speed and impaired balance compared to the control group (36), while Basford et al. (11) report higher postural sway which were associated with sensory deficits in the peripheral vestibular system. These studies collectively highlight the relationship between visual, vestibular, and balance dysfunction and the resulting impact on mobility.

Of interest, although assessment of the peripheral vestibular system using the computerized dynamic visual acuity has shown higher loss of dynamic visual acuity in young adults (between 20 and 30 years of age) in the chronic stage of concussion compared to controls (21, 25), as well as in the previous mentioned studies (36, 57), no differences in dynamic visual acuity between the mTBI and control groups were seen in this study. Due to the high symptoms burden experienced by this population, which can be increased by head movement, we used the standard protocol that maintains a head velocity between 80 and 120 degrees/second in the yaw plane and between 60 and 100 degrees/second in the pitch plane, which should be adequate to detect peripheral vestibular hypofunction. Due to the small sample size we did not analyze participants who had >0.2 LogMAR loss in dynamic visual acuity (which is considered clinically abnormal). Future studies with larger sample sizes are necessary to examine the relationship between functional measures of gaze stability and functional mobility.

Like other studies, we observed an association between poorer dynamic balance and higher symptom severity (9, 15, 23, 36, 57, 67, 68). Dizziness is one of the most common symptoms experienced after mTBI, with close to 81% complaining of dizziness right after injury (69, 70). In our study, the DHI score in individuals with mTBI was highly variable with a range between 10 and 90. Higher perception of handicap due to dizziness/imbalance were associated with lower FGA performance scores. Additionally, higher scores on each subscale of the DHI; physical, functional, and emotional were associated with poorer FGA performance. Within the physical subscale, participants reported that performing strenuous leisure activities were difficult which aligns with results of the study by Bier et al. where leisure activities were shown to be less frequent and less strenuous compared to pre-mTBI injury status (13). Quick movements and bending down activities were also difficult for people with mTBI which is seen in their performance while walking quickly with head turns and tilts on the FGA. Within the functional subscale, participants reported that they had difficulty walking around the house in the dark. The item on the FGA that examines walking with eyes closed showed significantly lower scores in individuals with mTBI compared to control subjects. Kleffelgaard et al. (57) have reported that dizziness-related disability was predicted by pre-injury comorbidities, and was associated with more vertigo symptoms, balance problems, and psychological distress, showing that the relationship between perception of handicap and dynamic balance can be bidirectional. Likewise, the relationship between dizziness and emotional status including anxiety is complicated, bidirectional in nature, with high levels of anxiety resulting in deleterious effects on balance control (71–74).

We show an association between dynamic balance and post-concussion symptom severity. Kleffelgaard et al. (9) examined older adults with mTBI 4 years after injury and reported slower walking speed, poor balance, and lower Dynamic Gait Index scores which were associated with higher PCSS scores. We report similar findings where lower dynamic mobility scores were associated with higher PCSS scores while Fino and group report slower turning speed were correlated with symptoms such as headache, nausea, vision problems, and sensitivity to noise/light (36). The subscales of the PCSS that were associated with FGA performance were somatic complaints, cognitive, and sleep symptoms. Somatic symptoms (headache, nausea, vomiting, balance problems, dizziness, fatigue, light sensitivity, noise sensitivity, numbness, visual problems) can individually or collectively affect dynamic balance (75–78). In this study, two participants with mTBI had a past history of migraines, but 20/22 (90%, 1 person with missing data) had current symptoms of headache with 6 individuals reporting headache severity of 5 or 6. Cognitive symptoms (feeling slow, feeling foggy, difficulty concentrating, and difficulty remembering) are commonly seen after mTBI and can be related to impairments in executive function, visual attention, memory, information processing among others (79–81). Cognition is closely related to the vestibular system (82–85), in fact, motor training programs that incorporate cognitive tasks have shown a high success rate in increasing gait speed, reducing fall risk, and enhancing quality of life (86–88). Sleep quality and duration are affected after mTBI (89–91), and poor sleep habits can increase symptoms of dizziness (71), and affect balance adversely (92).

Results of this study have important implications for rehabilitation clinicians who treat people after mTBI. Due to persistent impairments and symptoms, patients may not feel confident continuing with daily activities resulting in lower activity levels, physical deconditioning, and social isolation. The FGA is a test that can be easily performed in the clinic setting to identify dynamic balance issues as well as to track recovery as symptoms subside with rehabilitation exercises. Future studies examining the effects of impaired balance and persistent symptoms on physical activity levels in chronic mTBI are necessary. Results of this study show that dynamic balance deficits continue to persist in chronic mTBI, hence, rehabilitation to address persisting deficits using strategies that are engaging should be considered. Exercising in a game format could increase engagement while providing feedback to patients regarding the accuracy of their performance. Use of virtual reality to encourage motor and cognitive pairing while simulating outdoor environments can provide more realistic models of training. If exercises by themselves are not adequate to induce neuroplasticity in the chronic stage of mTBI, pairing neuromodulation with exercise strategies may provide opportunities to improve functional outcomes (93).

### Limitations

This pilot study is not without limitations. Assessment of central oculomotor function was not performed using video-oculographic systems, hence, future studies that perform quantitative assessments of oculomotor performance are needed to be able to understand the relationship between impaired pathways and dynamic balance control after mTBI. Although our sample size was adequate to detect meaningful differences in mobility performance and symptom scores between people with mTBI and healthy controls, it was not large enough to analyze the role of medications or the effect of co-morbidities like diabetes that can affect mobility. We did not collect history of alcohol/ drug abuse, or psychological issues, and acknowledge that personal factors may impede recovery. Medical history was not verified in the control participants, history was collected by self-report. Although the age range for the study was 40–80 years, we mainly recruited individuals in the middle-aged range, which narrows the generalizability of the study.

## Conclusions

Dynamic balance deficits during dynamic mobility tasks persist in individuals with chronic mTBI. Oculomotor deficits, perception of handicap due to dizziness, and post-concussion symptoms are associated with poorer performance on the Functional Gait Assessment, a test that can be completed easily in the clinic setting. Interdisciplinary and multifaceted interventions that simultaneously address these deficits can improve outcomes.

## Data Availability Statement

The raw data supporting the conclusions of this article will be made available by the authors, without undue reservation.

## Ethics Statement

The studies involving human participants were reviewed and approved by Institutional Review Board at the University of Kansas Medical Center. The patients/participants provided their written informed consent to participate in this study.

## Author Contributions

LD'S: conception of study, data collection, data analysis, writing, reviewing, and revising of manuscript. PC and HD: conception of study, data analysis, writing, reviewing, and revising of manuscript. SO: data collection, data entry and analysis, reviewing, and revising of manuscript. MR: recruitment for study, reviewing, and revising of manuscript. All authors contributed to the article and approved the submitted version.

## Conflict of Interest

The authors declare that the research was conducted in the absence of any commercial or financial relationships that could be construed as a potential conflict of interest.

## Publisher's Note

All claims expressed in this article are solely those of the authors and do not necessarily represent those of their affiliated organizations, or those of the publisher, the editors and the reviewers. Any product that may be evaluated in this article, or claim that may be made by its manufacturer, is not guaranteed or endorsed by the publisher.
